# Effects of Pineapple Peel Ethanolic Extract on the Physicochemical and Textural Properties of Surimi Prepared from Silver Carp (*Hypophthalmichthys molitrix*)

**DOI:** 10.3390/foods11203223

**Published:** 2022-10-15

**Authors:** Sanjeev Sharma, Ranendra Kumar Majumdar, Naresh Kumar Mehta, Nilesh Prakash Nirmal

**Affiliations:** 1 College of Fisheries, Central Agricultural University (Imphal), Lembucherra, Imphal 799210, Tripura, India; 2 Institute of Nutrition, Mahidol University, 999 Phutthamonthon 4 Road, Salaya 73170, Thailand

**Keywords:** surimi, silver carp, pineapple peel, phenolic compounds, gelation

## Abstract

The effects of ethanolic pineapple peel extract (PPE) powder at various concentrations (0–1.50%, *w*/*w*) on the gelling properties of silver carp surimi were investigated. The pineapple peel extract produced with 0–100% ethanol, revealed that 100% ethanol had the highest bioactive properties. Surimi gels with added PPE powder demonstrated improved gel strength (504.13 ± 11.78 g.cm) and breaking force (511.64 ± 11.80 g) up to 1% PPE addition; however, as PPE concentration increased beyond 1%, the gel strength decreased. Similarly, with the addition of 1% PPE powder, more hydrophobic bonds and fewer sulfhydryl groups and free amino groups were seen. However, the gels with PPE powder added showed a slight reduction in the whiteness of the surimi gels. FTIR analysis indicated that the fortification with PPE powder brought about the secondary structure of myofibrillar proteins; peaks shifted to the β-sheet region (PPE gels) from the α-helix region (control). SEM analysis indicated that the gel with 1% PPE powder had a relatively organized, finer and denser gel architecture. Overall results suggested that the addition of PPE powder up to 1% to the surimi gels enhanced the gelling properties as well as the microstructure of the surimi.

## 1. Introduction

The agro-industrial processing sector generates different by-products which are abundant in numerous health benefit compounds [1]. Among different types of agro waste, fruit peelings are considered as the major by-product. The recovery of functional molecules from these by-products may find applications in the food sector and thereby decrease the amount of waste deposition. Pineapple (*Ananas comosus*) is the second most consumed and produced fruit after banana, accounting for roughly 20% of total tropical fruit production [2]. The pineapple canneries where approximately 75% of the fruit is discarded as waste, causing problems of disposal and pollution [3], could be a source of valuable bioactive compounds for beneficial uses.

Phenolics are secondary metabolites produced through the shikimic acid, malonate–acetate, and isoprenoid pathways in plants. These compounds comprise one or more aromatic rings with hydroxyl (-OH) groups [4]. Polyphenol species have a great range of biological and pharmacological actions, with antioxidant, anti-inflammatory, anti-obesity, antidiabetic, anticancer, antibacterial, antiallergic, and hepatoprotective properties due to their unique molecular structures [5,6]. Protein–polyphenol conjugates are formed primarily by hydrogen bonding and hydrophobic–hydrophobic interactions [7,8]. Hydrogen bonds are formed when the -OH groups of phenolics interact with oxygen or nitrogen, specifically the -OH and amino groups of proteins [9,10], while covalent bonds are characterized by the construction of irreversible interactions under alkaline conditions or the presence of phenolic oxidases [10]. Dominant phenolics present in the pineapple peel extract include gallic acid, epicatechin, catechin, and ferulic acid [11]. The main dietary phenolic components include phenolic acids, flavonoids, and tannins, which have a variety of health benefits. Pineapple peel extract shows an appreciable antioxidant activity including DPPH, FRAP and ABTS scavenging activity [12], anti-inflammatory, antiviral, antiparasitic, and antifungal properties [13]. Punbusayakul et al. [14] reported the antimicrobial activity of pineapple peel against some food-borne pathogens (*Bacillus cereus*, *Salmonella typhimurium*, *Staphylococcus aureus*, and *Escherichia coli*).

Surimi, a water-washed fish minced meat, rich in myofibrillar proteins, devoid of impurities, exhibits peculiar textural features and gelling properties, and hence, is often consumed as a value-added product including fish balls, mimic crab sticks, fish tofu, etc., [15,16]. For surimi-based goods, gelation is a key stage in achieving the required texture, and myofibrillar proteins play a key role in the disulfide and non-disulfide covalent bonding that crosslinks surimi gel to form a three-dimensional protein network [17]. Silver carp (*Hypophthalmichthys molitrix*) is presently being considered as an alternative raw material for surimi manufacturing as a result of deep processing and dwindling marine fish resources. Because of its inexpensiveness, rapid growth, excellent nutritional content, and white meat color, silver carp is a popular freshwater fish [16]. However, the overall quality of surimi prepared from freshwater fish species is not so pleasing due to its lower gelling ability and acceptability. Some studies have been carried out using several food grade ingredients, viz., bovine plasma serum, egg white and microbial transglutaminase (MTGase) as gel enhancer. However, most of them adversely affected the microstructure as well as the sensory properties of the surimi [17,18,19]. Polyphenols are widely known for their ability to bind well with protein; as a result, scientists believe polyphenols from fruit or vegetable waste could significantly improve the gel strength as well as the nutritional value of the surimi [10,17,18,19]. The nature of noncovalent (hydrogen, hydrophobic and van der Waals forces) or covalent bonds and their interaction with polyphenols contribute to changes in the functional properties of proteins [10]. Therefore, this study focused on the effects of pineapple peel extract powder on the gelling ability, textural profiles, color, chemical interactions as well as the gel microstructure of surimi from silver carp.

## 2. Materials and Methods

### 2.1. Materials

Fresh silver carp (*Hypophthalmichthys molitrix*) weighing 1.5 ± 0.10 kg and length 50 ± 2.2 cm was procured from Lake Chowmuni fish market, Agartala, India. The experimental fish were transported in a thermocol box with ice to the fish processing facility of the College of Fisheries, (CAU-I), Lembucherra, Agartala, Tripura, India, within half an hour. The peel of ripened pineapples was collected from fruit processing centers of Agartala, Tripura. The peel (average thickness 5 mm) was yellow-green in color having rough outer surface and inner surface with little adherent flesh.

### 2.2. Pineapple Peel Extract (PPE) Preparation

The PPE was prepared as per the method described by Malviya et al. [20] with slight modifications. Pineapple peel was washed with tap water and subjected to drying in a hot air oven at 50 ± 2 °C up to a moisture content of nearly 10%. The dried peel was pounded to a coarse powder using a mixer grinder at low speed (10,000–12,000 rpm), riddled with an 80-mesh size sieve and finally kept in the deep freezer (−18 °C) until further use. The PP powder was extracted with different concentrations of ethanol (0, 40, 60, 80, and 100%) with the ratio of 20 g powder in 100 mL extraction solvent. The samples were extracted for 24 h at 37 °C using a shaker incubator (Labtech, Jaipur, India) at 200 rpm. Then, suspensions were filtered using Whatman no. 1 and the filtrate was kept at 4 °C in the incubator. The process was repeated three times and the pooled filtrate was concentrated using vacuum rotary evaporator (J.P. Selecta, Barcelona, Spain). Consequently, the concentrate was lyophilized using laboratory freeze dryer (Thermo Electron Corp., Waltham, MA, USA) to obtain extract powder, which was used for further experiments. The extract yield was calculated using the formula:

(1) $$\mathrm{Yield}\;\left(\%\right)=\frac{\mathrm{Weight}\;\mathrm{of}\;\mathrm{the}\;\mathrm{freeze}-\mathrm{dried}\;\mathrm{extract}}{\mathrm{Weight}\;\mathrm{of}\;\mathrm{the}\;\mathrm{dry}\;\mathrm{ground}\;\mathrm{peel}}\times 100$$

#### 2.2.1. Total Phenolics Content, Flavonoids Content, and Tannin Contents Determination

Total phenolic content (TPC) of pineapple peel extract was determined using Folin-Ciocalteau Reagent (FCR) as described by Buamard and Benjakul [18] with a slight modification. The 100 μL of prediluted extract with distilled water (mg/mL) was mixed with 0.75 mL of FCR. After 5 min, the reaction was added with 0.75 mL of 6% (*v*/*v*) sodium carbonate. The solution was mixed well and allowed to stand for 1 h at room temperature. The absorbance at 760 nm was recorded using UV–vis spectrophotometer. Standard solutions of gallic acid (0–100 ppm) were used for standard curve preparation. The phenolic content was expressed as mg Gallic acid equivalents (GAE) per 100 g dry weight of pineapple peel extract.

Total flavonoid content (TFC) was determined following the modified procedure of Zhishen et al. [21]. A sample of 1 mL of diluted peel extracts with water (*w*/*v*, mg/mL) was placed in a 5 mL volumetric flask, then 200 μL of distilled water were mixed, followed by 0.15 mL of 5% NaNO_2_. Next, after 5 min, 0.15 mL of 10% AlCl_3_ was added. Then 5 min later, 1 mL of 1 M NaOH was added and the volume made up to 5 mL with distilled water. The solution was mixed gently and absorbance was measured at 510 nm using a spectrophotometer. Standard solutions of quercetin (0–600 μg/mL) were used for standard curve preparation. Total flavonoid contents were expressed as mg quercetin equivalent (QE) g^−1^ of dry peel extracts.

Total tannin content (TTC) in the dried peel extracts was determined by Folin–Ciocalteu method as suggested by Haile and Kang [22]. About 0.1 mL of prediluted extracts (mg/mL) was added to a volumetric flask (10 mL) containing 7.5 mL of distilled water and 0.5 mL of Folin–Ciocalteu phenol reagent; 1 mL of 35% sodium carbonate solution was poured in and finally diluted to 10 mL with distilled water. The mixture was shaken well and kept at room temperature for 30 min. A set of reference standard solutions of tannic acid (0, 20, 40, 60, 80, 100 µg/mL) were prepared. The absorbance for samples and standard solutions were measured at 700 nm with an UV–visible spectrophotometer. The estimation of the tannin content was carried out in triplicate. The tannin content was expressed in terms of mg tannic acid equivalents (TAE) per g of dried sample.

#### 2.2.2. Antioxidant Activity Determination

The 2,2-Diphenyl-1-picrylhydrazyl (DPPH) radical scavenging activity of peel extracts was determined according to the method of Brand-Williams et al. [23] with some modifications. The stock solution was prepared by dissolving 24 mg DPPH with 100 mL methanol and then stored at −20 °C until further use. The working solution was obtained by mixing 10 mL stock solution with 45 mL methanol. The 150 μL prediluted extract (mg/mL in water) was mixed with 2850 μL of DPPH working solution and left for 24 h in the dark. Then, the absorbance was taken at 515 nm using a UV–visible spectrophotometer. The DPPH percentage was calculated by following equation:

(2) DPPH scavenging activity (%)=(A0−A1)/A0×100

where A0 = OD of control; A1 = OD of sample

For 2, 2′-Azino-Bis-3-Ethylbenzothiazoline-6-Sulfonic Acid (ABTS) assay, the method of Arnao et al. [24] with some modifications was followed in this study. The stock solution was prepared as 7.4 mM ABTS solution and 2.6 mM potassium persulfate solution separately. The working solution was then prepared by mixing the two stock solutions in equal quantities and allowing them to react for 12 h at room temperature in the dark. The working solution was then diluted by mixing 1 mL ABTS solution with 60 mL methanol to obtain an absorbance of 1.170 at 734 nm using the spectrophotometer. Fresh ABTS solution was prepared for each assay. Peel extracts (diluted as mg/mL) 150 µL were allowed to react with 2850 µL of the ABTS working solution for 2 h in dark conditions. Then the absorbance was taken at 734 nm using the spectrophotometer. The ABTS percentage was calculated by following equation

(3) ABTS scavenging activity (%)=(A0−A1)/A0×100

where A0 = OD of control; A1 = OD of sample

The ferric reducing antioxidant power (FRAP) assay [25] is based on the ability of phenolics to reduce Fe^+3^ to Fe^+2^. To prepare the FRAP reagent, 0.1 M acetate buffer (pH 3.6), 10 mM TPTZ, and 20 mM ferric chloride (10:01:01, *v*/*v*/*v*) were mixed together. A 20 µL measure of previously diluted extract with water was added to 150 µL of FRAP reagent and well-mixed samples were incubated at 37°C for 10 min. The absorbance was measured at 593 nm 25 in UV–visible spectrophotometer. The analysis was performed in triplicate, using an aqueous FeSO4 solution as standard and the results were expressed as micromole FeSO_4_ equivalents/g dry weight samples.

The PPE powders with the highest total polyphenols and antioxidant activities were used for further experiments.

### 2.3. Surimi Gel Preparation

Minced meat was washed three times consecutively for 10 min duration (twice with potable water followed by 0.1% NaCl solution to maximize dewatering). The minced meat to water ratio was 1:4 (*w*/*v*), maintaining the water temperature of 10 °C. The settling slurry was filtered using double-layer cheesecloth, squeezed manually, and then centrifuged to remove extra water using a basket centrifuge. Finally, the surimi at 500 g was packed in low density polyethylene pouches.

The surimi was mixed with selected PPE powder (100% ethanol extracted) at 0.0, 0.25, 0.50, 0.75, 1.00, 1.25 and 1.50 (*w*/*w*). The PPE-powder-added surimi was further mixed with 2.5% of NaCl followed by mixing in a mixer grinder to obtain a homogenous paste, and left at room temperature for one hour before stuffing. The paste was filled into vinylidene chloride casing (10 cm length, 2.0 cm diameter) followed by two-step thermal setting method, i.e., at 40 °C for 30 min and at 90 °C for 20 min, as described by Luo et al. [26]. Thereafter, gel casings were immediately cooled to 4–5 °C by immersing in ice water for 30 min. The cooled surimi gels were stored at 4 °C overnight and subjected to characterization.

#### 2.3.1. Surimi Gel Strength Determination

The surimi gels were brought to room temperature (28 °C) and casings were removed. The gels were sliced into cylinder-shaped samples with a sharp blade (2.5 cm in length). A texture analyzer (TA-XT PLUS, Stable Micro System Ltd., Surrey, UK) connected with a spherical plunger (diameter 5 mm, P/5 S) was used to determine characteristics of the gels such as breaking force and deformation. The instrumental parameters used during analyses are trigger force (10 g), test speed (1 mm s^−1^) and compression distance (15 mm), respectively. The gel strength of the surimi was calculated from breaking force (g) and deformation (cm) for each sample.

#### 2.3.2. Texture Profile Analysis (TPA)

Textural characteristics including hardness, adhesiveness, springiness, cohesiveness, gumminess and chewiness of all samples were determined using a texture analyzer (TA-XT PLUS, Stable Micro Systems Ltd., Surrey, UK) fitted with aluminum cylindrical probe (dia. 75 mm). The pre-test was set at 1.0 mm s^−1^, while post-test speeds were set at 5 mm s^−1^ and compression distance was conducted at 10 mm, respectively.

#### 2.3.3. Determination of Water Holding Capacity (WHC), Protein Solubility (PS) and pH

The WHC of surimi gels was evaluated in triplicate by following the procedure designed by Barrera et al. [27]. A 5 g portion of surimi gels was weighed and put onto 8 layers of filter paper (Whatman No. 1). Samples were placed at the bottom of 50 mL centrifuge tubes and centrifuged at 5000× *g* at 4 °C for 15 min (make REMI, Mumbai, India). Immediately after centrifugation, the gels were removed and re-weighed. The WHC was expressed as the weight of the centrifuged gels relative to the original weight of samples.

(4) WHC (%)=(W2/W1)×100

where W1 represents the weight of the gel before centrifugation and W2 represents the weight of the gel after centrifugation.

For the protein solubility test, 2 g of surimi gel samples were homogenized with 18 mL of 0.6 M KCl for 30 s, according to methods of Benjakul et al. [28]. The homogenate was stirred at room temperature i.e., 25–27 °C for 4 h, followed by centrifugation at 12,000× *g* for 20 min at 4 °C. To 10 mL of the supernatant, cold 50% (*w*/*v*) TCA was added to obtain the final concentration of 10%. The precipitate was washed with 10% TCA and solubilized in 0.5 M NaOH. Protein content was determined following Biuret method [29]. For estimation of total proteins, surimi was completely solubilized directly in 0.5 M NaOH.

Calculation:

(5) Protein solubility (%)=(Protein Concentration in supernatant/Total Protein)×100

To determine the pH of surimi gels, 5 g gel sample was homogenized with 50 mL distilled water for 30 s. The pH value of homogenate was recorded using a digital pH meter (Sartorius). Three readings were made for each of the 27 samples and the mean value was recorded. The pH meter was calibrated to pH 4.0 and 7.0 before each estimation.

#### 2.3.4. Determination of Chemical Interactions in Surimi Gels

Chemical interactions in surimi gel were determined using the method described by Arsyad et al. [30]. Briefly, a finely chopped 2 g gel sample was mixed with 10 mL of reaction solution (reaction solution contained 10 mL of each of 0.05 M NaCl (SA), 0.6 M NaCl (SB), 0.6 M NaCl and 1.5 M urea (SC) and 0.6 M NaCl and 8 M urea (SD)) and homogenized at 4500 rpm in tissue homogenizer (Ultra-Turrax, IKA, Königswinter, German) for 1 h. After that the mixture was centrifuged at 10,000× *g* (Centrifuge 5430 R, Eppendorf, Hamburg, Germany) for 15 min. The protein content of the supernatant was estimated following Biuret method [29] to determine the existence of ionic bonds (the difference between SB and SA), the hydrogen bonds (the difference between SC and SB) and hydrophobic interactions (differences between SD and SC).

#### 2.3.5. Determination of Total Sulfhydryl (SH) and Free Amino Groups of Natural Actomyosin of Gels

Natural actomyosin (NAM) was extracted from prepared surimi as per the method of Ogawa et al. [31]. After that, PPE powder (0.25 to 1.50% *w*/*v*) was added to 20 mL of NAM solution (3.5 mg/mL). The mixture was heated at 40 ± 1 °C for 30 min then at 90 ± 2 °C for 20 min. After heating, samples were cooled in iced water and subjected to sulfhydryl and free amino group determination using DTNB and o-phthaldialdehyde, respectively [31].

#### 2.3.6. Gel Whiteness Determination

The gel whiteness was determined using Spectrocolorimeter (ColorFlex EZ, Hunter Associates Laboratory, Inc., Reston, VA, USA). The color coordinates such as L* (blackness/whiteness), a* (redness/greenness) and b* (yellowness/blueness) were determined using CIE method [32], and the whiteness was calculated using the following equation.

Whiteness = 100 − [(100 − L*)^2^ + a*^2^ + b*^2^]^1/2^ (6)

#### 2.3.7. Fourier Transform Infrared Attenuated Total Reflection Spectroscopy (FTIR-ATR)

FTIR-ATR (ALPHA-FTIR, Bruker, Bremen, Germany) was used to evaluate the secondary protein structures of the gels. Freeze-dried surimi gel samples were used for FTIR spectrophotometer analysis at a resolution of 4 cm^−1^ and 32 scans at room temperature. Infrared spectra were recorded between 4000 and 500 cm^−1^. Spectral data were collected and baseline normalized by means of Bruker OPUS software version 7.0.

#### 2.3.8. Scanning Electron Microscope

Microstructure of prepared surimi gels were determined using scanning electron microscope (SEM) (ZEISS- Sigma 300, Aalen, Germany) as explained by Oujifard et al. [33].

### 2.4. Statistical Analysis

SPSS version 22.0 was used for statistical analysis (IBM SPSS, Chicago, IL, USA). All assays were carried out in triplicate. All data was examined with one-way analysis of variance (ANOVA) and expressed as standard deviation (SD). Duncan’s multiple range test was used to resolve the statistical difference between the control and treatment gels (*p* < 0.05).

## 3. Results and Discussion

### 3.1. Polyphenol Contents in PPE Powder

**Figure 1:**
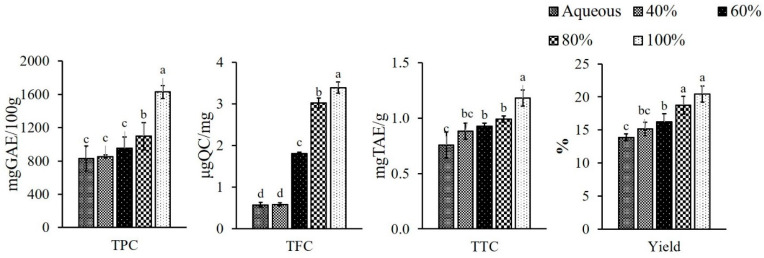
Phytochemicals in pineapple peel extracts (PPE) powder; where TPC—total phenolic content; TFC—total flavonoid content; TTC—total tannin content. Different letters (a, b, c, etc.) on the bars indicate significant differences (*p* < 0.05).

Extracts were prepared from pineapple peel using different concentrations of ethanol (40, 60, 80 and 100%, *v*/*v*) and aqueous medium to optimize the presence of maximum bioactive compounds, as illustrated in Figure 1. Extraction was performed in alcohol (ethanol), as it is an environmentally caring solvent [34], and which increases the amount of medium polar to polar components in the extract, primarily phenolic acids, flavonoids, sugars, and polysaccharides [35]. The ethanolic extracts showed significantly (*p* < 0.05) better yield compared to the aqueous one. Among the different ethanol concentrations, the maximum yield (*p* < 0.05) was obtained with 100% ethanol, i.e., 20.45% followed by 18.75, 16.22 and 15.14% with 80, 60 and 40%, respectively; whereas, the minimum yield was found to be 13.89% in aqueous extract. Li et al. [11] documented a higher yield (24.95%) when pineapple peel was extracted with methanol, while in another study, Hossain and Rahman [36] reported the higher yield in methanol (21.50%) followed by ethyl acetate (4.90%) and water extract (4.30%), which is concomitant with the current findings.

TPC of the extracts powder were 1628.63 ± 76.67, 1097.17 ± 163.89, 953.20 ± 134.23 and 853.57 ± 19.17 mg GAE/100 g of dry weight when extracted with 100, 80, 60 and 40% ethanol, respectively. The aqueous extract showed the lowest TPC as 831.43 ± 149.81 mg GAE/100 g. This may be explained by the phenolic compounds in the aqueous solvent becoming degraded due to the higher activity of enzyme polyphenol oxidase (PPO), and showing low efficiency, whereas these enzymes were inactivated in alcoholic (ethanol) media [37,38] resulting in higher yield of TPC. According to a recent study by Huang et al. [39], the pineapple waste yielded TPC in a range of 2080–3600 mgGAE/100 g and 1100–3000 mgGAE/100 g when extracted with ethanol and water, respectively, which corroborated the result of the current study. In another study, TPC was recorded as 1073 mgGAE/100 g crude extract with 50% ethanol [40] and 1110 ± 0.01 mgGAE/100 g when extracted with water [12]. Differences in the yield of TPC may be attributed to the kind of phenolic component present, as well as changes in the processing procedures and the solvents utilized [41].

The TFC of the extract powders ranged from 0.58 ± 0.04 to 3.39 ± 0.14 μgQC/mg of PPE powder and increased significantly (*p* < 0.05) with the increase in ethanol concentration, while the aqueous extract showed the value of 0.57 ± 0.06 μgQC/mg. TFC had a positive correlation with TPC (r = 0.850). Suleria et al. [42] found slightly higher values for TFC in ethanol at 1.47 ± 0.07 TFC (μgQC/mg) than that extracted with water at 1.35 ± 0.03 TFC (μgQC/mg). The variation in the TFC content may be attributed to the inherent variability of the raw material due to geographical conditions and applied methodologies [43]. The TTC increased significantly (*p* < 0.05) as the concentration of ethanol was increased and ranged from 0.88 ± 0.07 to 1.18 ± 0.07 mgTAE/g and the lowest was found in the aqueous extract (0.76 ± 0.12 mgTAE/g). The TTC had a similar trend to the TPC, showing a positive correlation (r = 0.785). The current study was well supported by the study of Suleria et al. [42] who reported an almost similar yield of TTC as 1.23 mgCE/g when extracted with ethanol.

### 3.2. Antioxidant Activities of the PPE Powder

**Figure 2:**
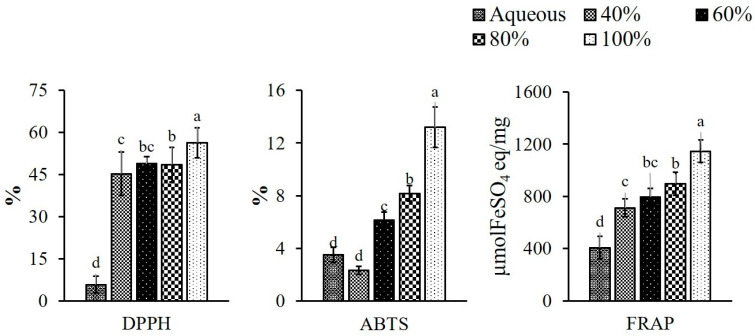
Antioxidative potentials of pineapple peel extracts (PPE); where DPPH—2,2-Diphenyl-1-picrylhydrazyl; ABTS—2, 2′-Azino-Bis-3-Ethylbenzothiazoline-6-Sulfonic Acid; FRAP—ferric reducing antioxidant power. Different letters (a, b, c, etc.) on the bars indicate significant differences (*p* < 0.05).

The antioxidant potential of the PPE powder, documented as DPPH, ABTS radical scavenging activities and FRAP, is presented in Figure 2. The DPPH free radical scavenging potentials of the extracts were found to be in the order of 100 > 80 > 60 > 40 (% ethanol concentration) > aqueous extract. This might be due to the fact that phenolic compounds are the primary contributors to plants’ antioxidant activity. Among the important biological properties exhibited by plant polyphenols, their antioxidant activity is of great interest. The effect of free radical scavenging activity of PPE on DPPH radicals is thought to be due to their hydrogen donation ability [44]. Some previous studies are also in support of the current one in that the antioxidant activity is correlated to phenolic content (r = 0.518) due to the ability of phenolics to donate hydrogen and stable radical intermediates [45,46]. Aa similar trend was observed in respect of ABTS radical scavenging activity, which ranged from 2.34 ± 0.31% to 13.19 ± 1.53% from 40 to 100%, whereas, the aqueous extract showed slightly higher activity (3.50 ± 0.57%) than the 40% of ethanolic extract. The ABTS activity of 1.30 ± 0.16% in pineapple peel was reported when extracted with 70% of ethanol [42]. Campos et al. [47] also found the ABTS radical scavenging ability in pineapple waste including stem and peel within the range of (2 and 12%) when extracted with methanol. This study stands with the current results. The differences in antioxidant activity in the different extracts can be explained by differences in the solubilization of the antioxidant component [48]. The ferric reducing antioxidant power of the pineapple peel extracts was in the range of 407.61 ± 87.41 to 1145.10 ± 86.00 μmolFeSO_4_ eq/mg with the lowest and the highest values in aqueous and 100% ethanol extract, respectively. The increase in reducing power was attributed to the higher phenolic content of the extract [44]. The correlation (r) with DPPH and ABTS was 0.870 and 0.782, respectively. Alothman et al. [49] observed the FRAP in pineapple waste in a range of 2170, 3410 and 1720 μmolFeSO_4_ eq/mg when waste was extracted with 50, 70 and 90% ethanol, respectively, whereas aqueous extract showed the value of 2230 μmolFeSO_4_ eq/mg. The current result of the lower FRAP value of pineapple peel extract may be explained by the fact that different parts of the fruit have distinct qualities, and also due to the influence of the extraction solvent and processing procedures used. The overall results indicated that the antioxidant activities of all extracts were related to their polyphenol content, where the increase in ethanol concentration showed increased phenolic content, which led to the higher antioxidant activities of PPE with the 100% ethanol extracted sample.

### 3.3. Effect of PPE Powder at Different Levels on Gel Properties of Surimi

#### 3.3.1. Effects on Textural Characteristics of Surimi

##### Breaking Force (BF), Deformation and Gel Strength

**Figure 3:**
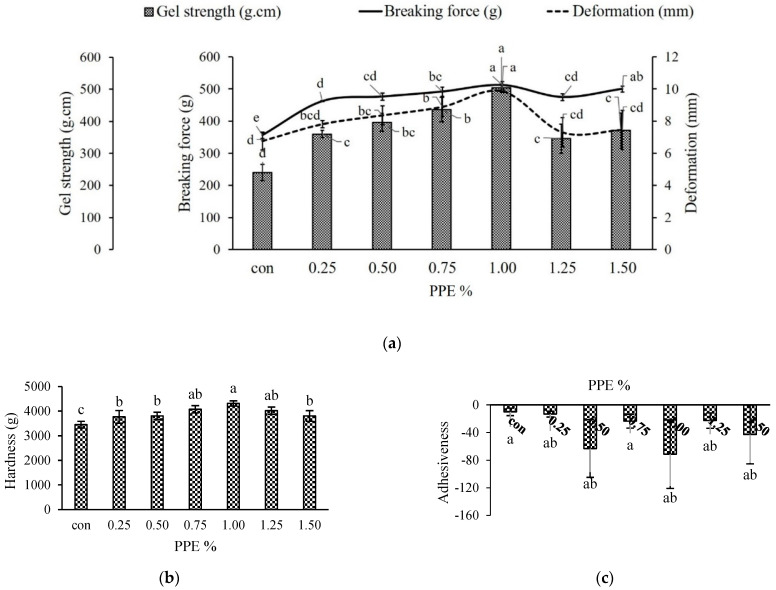

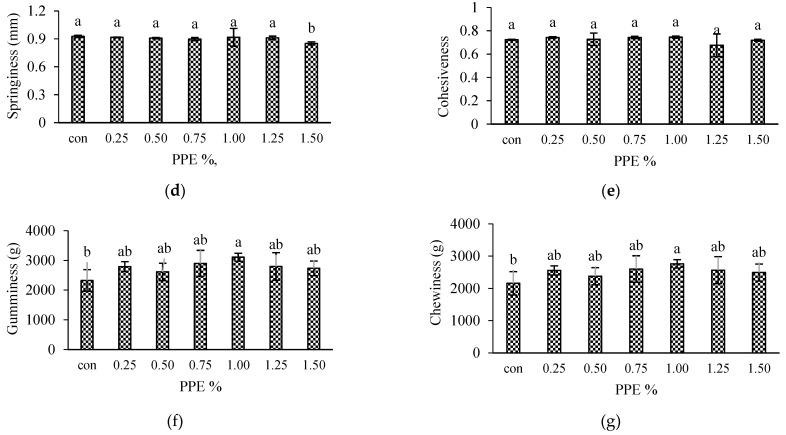
Textural properties of silver carp surimi gels with added PPE powder at different concentrations (0–1.5% *w*/*w*). Gel strength (**a**), hardness (**b**), adhesiveness (**c**), springiness (**d**), cohesiveness (**e**), gumminess (**f**) and chewiness (**g**); con (control)-without PPE. Different letters (a, b, c, etc.) on the bars indicate significant differences (*p* < 0.05).

The breaking force, deformation and gel strength of PPE-incorporated surimi gel from silver carp are displayed in Figure 3a. In the present investigation, a significantly (*p* < 0.05) higher breaking force (511.64 ± 11.80 g) was recorded with 1% PPE (%, *w*/*w*) whereas the control without PPE showed the lowest breaking force (355.71 ± 9.66 g). In treated gel samples, the breaking force increased significantly (*p* < 0.05) as the concentration of PPE increased up to 1%; although, beyond this concentration, the BF was found to be decreased significantly (*p* < 0.05). This may be attributed to the formation of noncovalent and covalent interactions between surimi protein and phenolics in PPE. In a similar study with coconut husk extracts, Buamard and Benjakul [18] reported that the hydrogen bonds, hydrophobic and covalent interactions were formed between myofibrillar protein and phenolic compounds of the extract. The phenolic compounds have a hydrogen donating ability that may establish hydrogen bonds with carboxyl groups of protein [50], resulting in the formation of greater cross-linking. When PPE is added to a surimi gel at higher concentrations, the excessive cross-linking may cause protein coagulation, probably due to the presence of the bromelain enzyme [37]. This excessive protein coagulation prevents formation of an ordered and fine gel network [38]. Self-aggregation of phenolic compounds at high levels of extracts could have resulted in a decrease in protein cross-linking capabilities [51]. As observed in this study, the phenolic compounds in the PPE at their optimum level were able to further enhance the breaking force with a resultant increase in the gel strength of the surimi.

The addition of various concentrations of PPE had a positive effect on deformation (mm), which significantly (*p* < 0.05) increased gradually up to 1% but afterwards decreased with 1.25 and 1.50% of PPE concentration. This was in similar line with breaking force (r = 0.576) and gel strength (r = 0.941). The results showed that PPE at different concentrations altered the native conformation of protein and gelation patterns, especially at 1% levels, facilitating more binding sites for phenolic–protein and protein–protein interactions at cooking temperatures which led to a strengthening of the gel networks [52]

While the deformation in gels containing 1.25 and 1.50% PPE showed a declining trend (*p* < 0.05), this may be the result of excessive aggregation.

Figure 3a illustrates the gel strength (g.cm) of PPE-added surimi gels as a combination of breaking force (g) and deformation (mm). In the current work, the gel strength of surimi with different concentrations of PPE was in the same line as with breaking force, where 1% PPE exhibited the highest (504.13 ± 11.78 g.cm) gel strength (*p* < 0.05), while the lowest value was recorded for control (240.29 ± 25.023 g.cm). The improved gel strength was most likely due to hydrophilic as well as hydrophobic interactions between polyphenols and the protein molecules of surimi [53]. The findings of the current work indicate that hydrogen bond and hydrophobic interactions are responsible for improve the gel strength by establishing the great phenolic–protein interactions. Nonetheless, covalent bonding may play a role to some extent. According to Balange and Benjakul [51], oxidized phenolic components can effectively boost the gel strength of mackerel surimi. As a result, 1% pineapple peel extract could be employed as a surimi gel-strength enhancer.

##### Texture Profile Analysis (TPA) of Surimi

Figure 3b–g depicts the effect of various levels of PPE on the textural attributes of surimi gels. Hardness is associated with the strength of the gel structure and is defined as the force required by the molars during the initial bite to split the sample into several fragments [54]. In the current investigation, the hardness increased significantly (*p* < 0.05) when PPE levels increased from 0.25 to 1% (Figure 3b), with 1% PPE having higher values (4311.54 ± 104.82 g) while control had the lowest values (3446.24 ± 136.27 g). PPE exhibited effects on gel hardness similar to those of BF (Figure 3a), and correlated well (r = 0.691). The findings showed that adding PPE to surimi gel could improve surimi gel strength (Figure 3a). Phenolic compounds polymerize and aggregate with myofibrillar protein during either setting or heating, leading to an increase in both breaking force and hardness, as reported by Arfat and Benjakul [52] while investigating the gel-forming ability of surimi from yellow stripe trevally prepared under different heating conditions. Petcharat and Benjakul [55] suggested that phenolic extracts of various types and forms can create protein–protein and phenolic–protein cross-linking in various modes, which can either depress or improve the main gel structure, depending on the extraction-media choice. A further decrease in hardness with higher PPE concentrations beyond 1% might be due to the masking of protein binding sites in the presence of a higher concentration of phenolic compounds which prevented protein–protein crosslinking; or the concentration of myofibrils was thinned by the addition of a higher level of extracts to the surimi gel with a consequent decrease in the breaking force leading to a weaker gel matrix [54]. Nevertheless, there were no significant (*p* > 0.05) variances in springiness (Figure 3d), elastic recovery that occurs when the compressive force is detached, and cohesiveness (Figure 3e), indicating the ability to break down the internal structure. Although the adhesiveness (Figure 3c) showed conflicting trends with varied PPE levels, Figure 3f,g demonstrates that PPE-fortified gels had significantly (*p* < 0.05) higher gumminess and chewiness values than the control, measured at 2325.69 ± 363.71 g and 2157.81 ± 362.66 g, respectively. However, among all the PPE samples, the 1% PPE gel had significantly (*p* < 0.05) higher values at 3107.02 ± 134.41 g and 2761.30 ±131.52 for gumminess and chewiness, respectively. Positive correlations were observed in both gumminess (r = 0.613) and chewiness (r = 0.544). Therefore, the addition of PPE at the optimum level could affect the textural properties of silver carp surimi gel.

#### 3.3.2. Water-Holding Capacity (WHC) of Surimi

**Figure 4:**
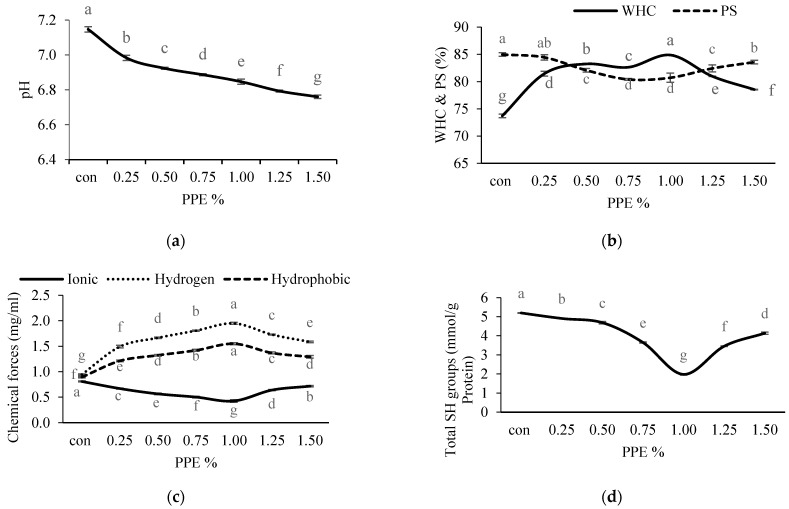
Effects of PPE powder at different concentration (0–1.5%, *w*/*w*) on the silver carp surimi gels pH (**a**), WHC (water holding capacity) and PS (protein solubility) (**b**), chemical forces (**c**), SH groups (**d**). Different letters (a, b, c, etc.) on the bars indicate significant differences (*p* < 0.05).

The WHC of surimi samples with PPE added at different concentrations is presented in Figure 4b. All the treated samples with different levels of PPE had significantly (*p* < 0.05) higher WHC compared to the control (without PPE) (73.73 ± 0.33%). Amongst all the treated samples, surimi gels with 1% of PPE showed a significantly (*p* < 0.05) higher value of WHC (84.89 ± 0.05%). The WHC of the PPE-added gels augmented significantly with the increase in PPE of up to 1% (*p* < 0.05), afterwards a decreasing trend was observed with an increasing concentration of PPE. Gel samples fortified with 1% PPE showed higher WHC, which was associated with a stronger breaking force (r = 0.821) and a higher ability to hold water (Figure 3a). During the setting process, phenolic extracts combine with denatured proteins to form an ordered network that can store more water [56]. In comparison with the control samples, the gel samples containing phenolic extract had a fine three-dimensional gel network that could adsorb or imbibe more water, as seen in the SEM pictures. The results of this study revealed that the phenolic compounds in PPE induced cross-linking with protein which led to a higher WHC up to an optimum PPE concentration of 1% and then decreased with PPE concentrations beyond this. A drop in WHC could be elucidated as an excessive aggregation of phenolic compounds and protein molecules narrowing the void spaces of the gel formed, which may result in a low WHC of the gel. Balange and Benjakul [51] obtained similar results when they treated the surimi gels with oxidized phenolic compounds.

#### 3.3.3. Protein Solubility (PS) of Surimi

The protein solubility values of the protein of surimi gels incorporated with PPE at different concentration are presented in Figure 4b. In this study, the solubility of incorporated gels slightly decreased significantly (*p* < 0.05) up to the optimum PPE concentration of 1% (80.41 ± 0.17%), then further increased significantly (*p* < 0.05) with the increasing concentration of PPE. The decrease in solubility implies the construction of protein aggregation, which could be due to quinone molecules’ cross-linking actions on proteins under setting and heating [17]. Greater breaking force and deformation were linked to a decline in solubility (Figure 3a). A previous study discovered that phenolic compounds and hydrophobic amino acids could establish hydrophobic interactions, which strengthened the gel matrix and reduced solubility [19]. Zhou et al. [57] observed the strong protein–protein interaction induced by phenolic compounds that were stabilized through covalent bonds such as S-S bonds. This was well evidenced by the decreasing free SH groups (Figure 4d) up to an optimum concentration of PPE.

#### 3.3.4. Chemical Interaction of Surimi

The influences of PPE on the chemical interactions of surimi gels are given in Figure 4c. Reed and Park [58] described the three forms of noncovalent chemical forces in surimi gel: hydrogen bonds, hydrophobic and ionic interactions. The greater number of hydrogen bonds in PPE-treated gel samples were observed compared to the control (without PPE), and this may be explained by the formation of additional hydrogen bonds between phenolic compounds and the amino acids of the protein [18]. Both gel strength and bound water stabilization (WHC) were enhanced mainly by hydrogen bonding (Figure 3a and Figure 4b). In this study, the hydrogen bonds increased significantly (*p* < 0.05) with the increase in PPE concentration of up to 1% (1.95 ± 0.02 mg/mL), and beyond this the hydrogen bonds decreased. The decrease in hydrogen bonds at a higher concentration of extracts could be related to the susceptibility of the phenolic–protein complex form at higher PPE concentration, during the heating process [59].

Ionic bonds are established by attractive Coulombic interactions between two amino acid residues with opposite charges, and they play a crucial role in the integrity of tertiary and quaternary configurations in proteins [60]. In the present study, the ionic strength in PPE-fortified surimi gels was found to be reduced significantly (*p* < 0.05) up to 1% (0.43 ± 0.03 mg/mL); beyond this concentration, the ionic strength was slightly increased. The ionic bonds and van der Waals force were disrupted during heating at 90 °C, and actin was largely attached to the gel matrix via hydrogen and hydrophobic interactions [61], resulting in the lowering of the ionic bonds. Moreover, the addition of PPE into the surimi gel enhances noncovalent interactions compared to ionic interactions. Additionally, the positive charges on the surimi protein surface were neutralized by negatively charged PPE resulting in a reduction of the ionic interactions because the addition of PPE significantly decreased the pH (Figure 4a) of the gel which ruptures the ionic interactions (r = 0.452).

For all the treated surimi gels, the hydrophobic bonds were observed as higher than the control (1.10 ± 0.02 mg/mL), and among the PPE-treated gels, 1% PPE exhibited significantly (*p* < 0.05) higher hydrophobic interaction (1.55 ± 0.02 mg/mL). Although, after increasing hydrophobic bonds up to 1% with increasing PPE concentration, a slight decreasing trend was found with the increase in PPE. During heating, the residues of hydrophobic amino acids were exposed through the unfolding of proteins, which strongly interact with the phenolic components of PPE, leading to increased protein crosslinking. Reed and Park [58] described that hydrophobic domain exposure during denaturation of protein contributes to a greater myosin aggregate, resulting in the formation of excellent elastic gels [62]. The key chemical forces that sustain the three-dimensional network structure against deformation (Figure 2a) are hydrophobic interactions and disulfide bonds [62]. A positive correlation between hydrophobic interactions and gel strength (r = 0.883) was noted. The affinity of protein to phenolic compounds increased as the concentration of PPE increased beyond 1%, leading to the hiding of the hydrophobic groups with a resultant lower bond availability.

#### 3.3.5. Total Sulfhydryl (SH) Groups and Free Amino Groups of Natural Actomyosin

The total SH group contents of surimi with or without PPE are depicted in Figure 4d. The control had significantly (*p* < 0.05) higher SH groups (5.20 ± 0.002 mmol/g protein) compared to PPE-treated samples. In PPE-treated NAM, the content decreased significantly (*p* < 0.05) with increasing concentrations of PPE up to 1%, which had the lowest value (1.96 ± 0.003 mmol/g protein), and thereafter SH content showed an increasing trend. In the presence of PPE during thermal gel setting, SH groups can be oxidized to disulfide bonds, which results in the development of protein cross-linking and improved gel strength, which is one explanation for the drop in SH groups in the treated NAM (Figure 2a). Similar findings were published by Cao et al. [63], who revealed that increasing the level of epigallocatechin gallate enhanced myofibrillar protein oxidation and resulted in the loss of SHs. Balange and Benjakul [17] also found that the SH contents were decreased with a corresponding increase in disulfide bonds during the addition of the phenolic compounds. In this study, it can be concluded that PPE at optimum concentration could change NAM configuration that renders the oxidation of sulfhydryl groups into disulfide bonds. Additionally, the reduction of SH groups was correlated with the solubility of protein (r = 0.794), which induced hydrophobic interactions. The further increase in SH groups beyond the optimum level of extracts may be due to a high quantity of quinones that can directly interact with SHs and mask [63] and protect them from oxidation loss [64].

**Figure 5:**
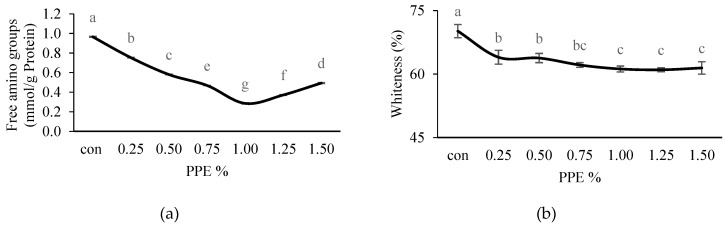
Effects of PPE powder at different concentration (0–1.5%, *w*/*w*) on the silver carp surimi gels free amino groups (**a**) and whiteness (**b**); con: control-without PPE. Different letters (a, b, c, etc.) on the bars indicate significant differences (*p* < 0.05).

The free amino group content of NAM from surimi silver carp, without and with different concentrations of PPE added (0.25–1.50%), was subjected to two-step heating as shown in Figure 5a. NAM samples without the addition of PPE (control) had a significantly (*p* < 0.05) higher value (0.97 ± 0.005 mmol/g protein) of free amino group content compared to the treated ones, whereas the treated samples showed a significant (*p* < 0.05) decreasing trend of free amino groups with increasing concentrations of PPE up to 1% (0.29 ± 0.005 mmol/g protein), but increased thereafter with PPE concentrations beyond 1%. During the heating process, the free amino groups of NAM could have been exposed to the phenolic compounds, resulting in lowered free amino contents [17]. A decreasing free amino group content indicates higher interactions between the phenolic compounds of the extracts (up to an optimum concentration) and protein side chains, causing higher cross-linking [65,66], which contributes to higher BF (r = −0.867) (Figure 3a). In contrast, the phenolic compound’s hydroxyl groups could be oxidized to form a free hydroxyl radical, which might react with oxygen or nitrogen from some amino acids, and also form hydrophobic interactions, causing oxidation followed by polymerization which subsequently lower the protein solubility [67]. In this study, an increase in the free amino group content of NAM, containing more than 1% PPE, may be attributed to the prevention of protein unfolding, due more to the interaction between the phenolics leading to protein aggregation than the phenolic–protein interactions, as suggested by the decreasing gel strength of the surimi.

#### 3.3.6. Whiteness of Surimi

The whiteness of silver carp surimi gels incorporated with different concentrations of PPE is displayed in Figure 5b. The current results showed that control (without PPE) had a significantly (*p* < 0.05) higher whiteness value (70.16 ± 1.56%), while for the PPE treated gels, the whiteness value significantly (*p* < 0.05) decreased gradually up to 1% (61.20 ± 0.70%), and did not change much thereafter. This was possibly due to the dark brown color of PPE. Sriket et al. [68] observed that fortification with phenolic compounds containing yanang leaf powder reduced the whiteness of fish sausage from tilapia (*Oreochromis niloticus*). However, further study is needed to improve the whiteness of the PPE-treated surimi gels.

#### 3.3.7. FTIR Spectra of Surimi

**Figure 6:**
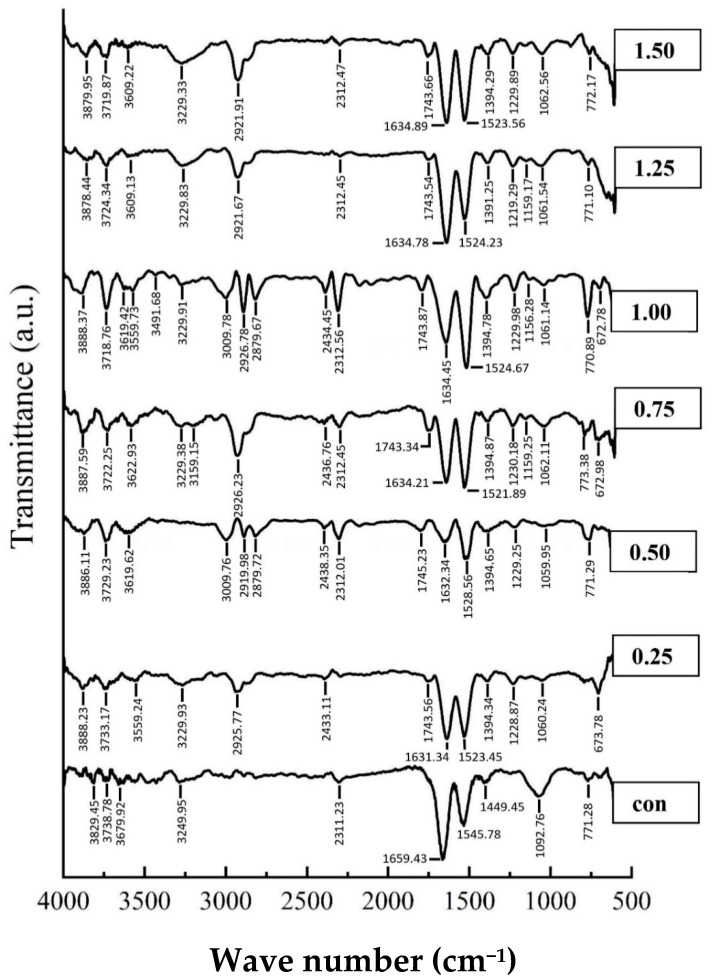
FTIR spectra of silver carp surimi gel with PPE powder added at different concentrations.

Figure 6 represents the FTIR spectra of surimi gels with and without PPE. The structural alterations of proteins are frequently determined using FTIR spectroscopy. The peak position at about the 1745 cm^−1^ wavenumber confirmed the successful melding of PPE and protein in the surimi gels, and it was assumed that the band formed from the stretching vibration of the C=O group in the phenolic compounds of PPE [69]. The amide I band (1600 and 1700 cm^−1^) is particularly valuable for the infrared spectroscopy study of protein secondary structures [70]. In general, the amide I band of the FTIR spectrum (amide I, signifying C=O stretching/hydrogen bonding associated with COO) has been categorized into **α**-helix (1658–1650 cm^−1^), **β**-sheet (1640–1615 cm^−1^), **β**-turn (1700–1660 cm^−1^), and random coil (1650–1640 cm^−1^) [15]. Furthermore, the structures of protein–polyphenol conjugates are determined using amide II (1480–1575 cm^−1^; N-H bending and C-N stretching), amide III (1229–1301 cm^−1^; C-N stretching and N–H bending), amide A (3300 cm^−1^; N-H stretching) and amide B (3000–2900 cm^−1^; C-H stretching and NH_3_) [10,15]. In the present study, it could be observed that a peak at 1657.43 cm^−1^ indicates the α-helix in the control shifted to the range of 1630–1635 cm^−1^ (β-sheet) in all treated gels, similarly, amide II bands were shifted from 1545.78 cm^−1^ (control) to lower wavenumbers 1523–1529 cm^−1^ in the PPE-added gels. The current results suggest that shifting of the α-helical peak to the β-sheet peaks could be due to alterations in the protein configuration upon polyphenol complexation [71]. The shifting of the α-helix structure to a β-sheet, which is related with the gelation mechanism, is expected to be the cause of the amide I band alteration [72]. The current results were in line with those of Feng et al. [73]. Similarly, Fan et al. [74] reported that the interaction between proteins and polyphenols caused reductions in amide (especially α-helix) and amide II wavenumber. Furthermore, PPE treated gels showed higher intensity at wavenumbers of 1075 and 1400 cm^−1^, which could be attributed to the C=O ester group stretching vibration and the bending of O-H bonds, respectively [54,72]. The greater amplitude, shown with the higher concentration of PPE fortification, could provide the OH group.

In addition, between 3200 and 3300 cm^−1^, an amide-A signal was discovered, indicating NH-stretching and hydrogen bonding; the results displayed that the amplitudes of this peak were higher in PPE-fortified gels (specially in 1% concentration of PPE) compared to control (without PPE). It could be associated with the quantity of the bonds (e.g., C-N and C-S bonds) between the incorporated phenolic compounds of PPE and the myofibrillar protein. This result is consistent with the formation of hydrogen bonds between the protein and polyphenol molecules [75]. Similarly, between wave lengths of 3000 and 2900 cm^−1^, PPE-fortified gels showed the highest intensity of amide B, which corresponded to the aliphatic CHs’ symmetrical and asymmetrical stretching vibrations in the CH_3_ and CH_2_ groups, respectively [72,76]. The phenolic compounds present in the extracts contributed to the formation of amide bonds with the protein of the gels [75]. As a result, adding PPE (especially 1% PPE) changed the secondary structure of the proteins, which in turn influenced the textural properties of the surimi.

#### 3.3.8. Microstructure of Surimi

**Figure 7:**
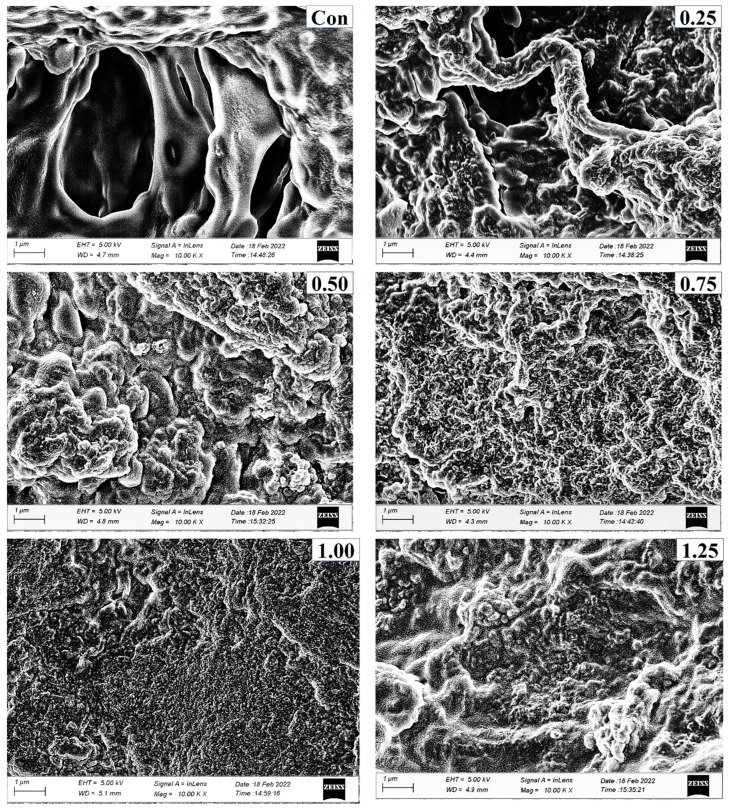

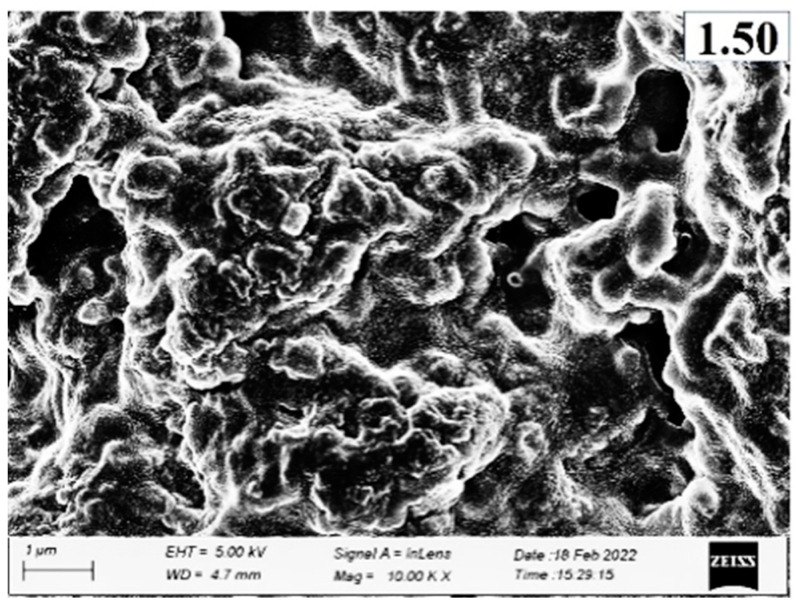
Electron microscopic images of surimi gels with different concentrations of PPE added (0.25–1.5%, *w*/*w*) at magnification of 10,000×. (Con) surimi without PPE; (0.25) surimi with 0.25% PPE added; (0.50) surimi with 0.50% PPE added; (0.75) surimi with 0.75% PPE added; (1.00) surimi with 1.00% PPE added; (1.25) surimi with 1.25% PPE added; and (1.50) surimi with 1.50% PPE added.

Microstructures of surimi gels incorporated with or without PPE are depicted in Figure 7. Larger voids or cavities with a coarser network were observed in the gel sample (control) without PPE addition compared to the fortified gels with PPE. This was correlated with the lower breaking force and WHC of the control. The PPE-fortified samples (particularly 1% PPE) had the lowest amount of voids or holes, the most regular, dense, and fine network. This was in line with a higher breaking force (Figure 3a), hardness (Figure 2b), and WHC (Figure 4b). This may be explained by the fact that during the heating process in the presence of phenolic compounds from PPE, especially at 1% of PPE concentration, a compact network was built through intermolecular cross-linking in conjunction with protein aggregation via hydrophobic, hydrogen bonds, and disulfide bonds (Figure 4c), which improved the WHC and gel strength [16]. With further increase in PPE concentrations beyond 1% PPE in gels, a larger and irregular microstructure was observed. This meant that phenolics from higher concentrations of PPE were filling protein fibers, resulting in the construction of an uneven gel network. As a result, the breaking force of the gel was reduced [56]. Additionally, a fine network gel with smaller holes can hold more water, which corresponds to the increased WHC of gels that were treated with PPE. As a result, PPE could enhance the three-dimensional network by improving the connection and organization at the optimal concentration (1%, *w*/*w*), leading to improved textural qualities. 

## 4. Conclusions

In comparison with water and aqueous ethanol concentrations (40, 60, and 80%), pineapple peel extract produced with pure ethanol showed greater phenolic content and antioxidant activity. Further, the addition of PPE powder at various concentrations (0.25−1.5% *w*/*w*) to the silver carp surimi gels affected the textural characteristics of the surimi. The addition of 1% PPE powder to the surimi gel produced superior textural properties compared to the other treatments. The WHC of the surimi slightly decreased with a higher concentration of PPE (1.25 and 1.5%). Furthermore, the whiteness of the surimi was found to be decreased with the increasing concentration of PPE (*p* < 0.05). Lowering of the pH upon addition of PPE powder significantly altered the chemical interactions which led to changes in the textural attributes of the gel. The oxidation of sulfhydryl groups to disulfide bonds during thermal treatment in the PPE-added gels resulted in a stronger gel network through protein crosslinking. Moreover, higher concentrations of PPE caused more phenolic–phenolic aggregation than protein–phenolic interaction resulting in a weak gel structure. The efficient blending of the myofibrillar protein and phenolic compounds found in PPE-treated surimi gels was validated by FTIR analysis. The SEM analysis revealed that a 1% PPE-powder addition to the surimi produced an ordered, finer and denser gel network compared to the other concentrations. Finally, the study suggested that adding PPE powder at a certain concentration (1%) can improve the textural characteristics of surimi. However, further study would be required to improve the whiteness of the gel.

## Data Availability

Data are available on reasonable request from the corresponding author.

## Novelty Impact Statement

Gelling is an important technological property of surimi, which has poor performance in the case of freshwater fish compared to marine water fish species. Depleted marine catches have forced the fish industry and/or researchers to find new candidate fish species without the addition of chemical gel enhancers. Therefore, this study unraveled the utilization of pineapple peel as a green additive to improve the gelling and textural properties of freshwater fish (silver carp). The outcome of this study will open up new horizons for the use of this species and pineapple peel extract for fish surimi preparation.
